# Proteomics-based evaluation of the mechanism underlying vascular injury via DNA interstrand crosslinks, glutathione perturbation, mitogen-activated protein kinase, and Wnt and ErbB signaling pathways induced by crotonaldehyde

**DOI:** 10.1186/s12014-022-09369-7

**Published:** 2022-08-24

**Authors:** Ming-Zhang Xie, Jun-Li Liu, Qing-Zu Gao, De-Ying Bo, Lei Wang, Xiao-Chun Zhou, Meng-Meng Zhao, Yu-Chao Zhang, Yu-Jing Zhang, Guo-An Zhao, Lu-Yang Jiao

**Affiliations:** 1 Department of Genetics, First Affiliated Hospital of Xinxiang Medical University, Xinxiang, 453000 Henan China; 2 Henan Key Laboratory of Neurorestoratology, Henan International Joint Laboratory of Neurorestoratology for Senile Dementia, The First Affiliated Hospital of Xinxiang Medical University, Weihui, 453100 Henan People’s Republic of China; 3 Department of Pathology, First Affiliated Hospital of Xinxiang Medical University, Xinxiang, 453000 Henan China; 4 Department of Laboratory, First Affiliated Hospital of Xinxiang Medical University, Xinxiang, 453000 Henan China; 5 Department of Cardiovascular, First Affiliated Hospital of Xinxiang Medical University, Xinxiang, 453000 Henan China

**Keywords:** Crotonaldehyde, Vascular injury, Differentially expressed proteins, Indicators, Wnt and ErbB signaling pathways

## Abstract

Crotonaldehyde (CRA)—one of the major environmental pollutants from tobacco smoke and industrial pollution—is associated with vascular injury (VI). We used proteomics to systematically characterize the presently unclear molecular mechanism of VI and to identify new related targets or signaling pathways after exposure to CRA. Cell survival assays were used to assess DNA damage, whereas oxidative stress was determined using colorimetric assays and by quantitative fluorescence study; additionally, cyclooxygenase-2, mitogen-activated protein kinase pathways, Wnt3a, β-catenin, phospho-ErbB2, and phospho-ErbB4 were assessed using ELISA. Proteins were quantitated via tandem mass tag-based liquid chromatography-mass spectrometry and bioinformatics analyses, and 34 differentially expressed proteins were confirmed using parallel reaction monitoring, which were defined as new indicators related to the mechanism underlying DNA damage; glutathione perturbation; mitogen-activated protein kinase; and the Wnt and ErbB signaling pathways in VI based on Gene Ontology, Kyoto Encyclopedia of Genes and Genomes, and protein–protein interaction network analyses. Parallel reaction monitoring confirmed significant (p < 0.05) upregulation (> 1.5-fold change) of 23 proteins and downregulation (< 0.667-fold change) of 11. The mechanisms of DNA interstrand crosslinks; glutathione perturbation; mitogen-activated protein kinase; cyclooxygenase-2; and the Wnt and ErbB signaling pathways may contribute to VI through their roles in DNA damage, oxidative stress, inflammation, vascular dysfunction, endothelial dysfunction, vascular remodeling, coagulation cascade, and the newly determined signaling pathways. Moreover, the Wnt and ErbB signaling pathways were identified as new disease pathways involved in VI. Taken together, the elucidated underlying mechanisms may help broaden existing understanding of the molecular mechanisms of VI induced by CRA.

**Supplementary Information:**

The online version contains supplementary material available at 10.1186/s12014-022-09369-7.

## Significance

Airborne pollutants play a major role in vascular injury-related morbidity and mortality. Of the well-known air pollutants, crotonaldehyde, one of the top nine pollutants from tobacco smoke, has been the subject of numerous studies aimed at determining the disease-inducing effects of exposure thereto. However, despite considerable interest on this subject, the molecular mechanisms underlying the effects have not been well investigated. Therefore, this research aimed to use proteomics techniques to determine the indicators and analyze the mechanism of DNA interstrand crosslinks; glutathione perturbation; mitogen-activated protein kinase; cyclooxygenase-2; and Wnt and ErbB signaling pathways, all of which may contribute to VI. Our results provide novel insights into the pathogenesis of crotonaldehyde-induced vascular injury. The significance of the research is highlighted by the growing importance of proteomics-based analyses of biological and pathophysiological processes to elucidate the mechanisms of disease development and to develop protein-based therapeutic approaches.

## Introduction

Crotonaldehyde (CRA)—a highly reactive α,β-unsaturated aldehyde—is a potent environmental pollutant; tobacco smoke is a major non-occupational source of exogenous CRA, which affects both passive and active smokers [1, 2]. CRA is considered as one of the top nine pollutants in cigarette smoke and is monitored by research groups mandated by the World Health Organization (WHO) to conduct surveillance on tobacco smoking globally [3]. It is also found in certain foods and beverages, including meat, vegetables, and wine [4]. Furthermore, CRA is an industrial pollutant arising from different chemical procedures or the synthesis of chemicals, such as tocopherols. Endogenous sources of CRA include lipid peroxidation and acetaldehyde metabolism [5]. Exposure to CRA via the respiratory tract, digestive tract, and close contact with the skin or eyes poses substantial threat and results in vascular injuries (VIs).

A previous study reported that CRA induced mutation in mitochondrial DNA and that acetylresveratrol can be used as a substitute for resveratrol to reverse the toxic effect of CRA [6]. Hypotension—an underappreciated cardiovascular risk factor for syncope—results after chronic or acute exposure to CRA, which induces an unconventional form of vascular/endothelium dysfunction [7]. CRA may affect dopaminergic pathways through inhibition of the conversion of dopamine to the metabolites homovanillic acid and 5-hydroxyindole acetic acid by monoamine oxidase [8], while CRA-induced HO-1 expression protects against oxidative stress in human umbilical vein endothelial cells [9]. These previous studies focused on functional impairment, such as oxidative stress.

Though biological dysfunctions of the vasculature are determined partly using traditional methods, such as colorimetric assays and quantitative fluorescence [7–10], there are several key mechanisms underlying VI that are difficult to investigate using traditional methods. Aldehydes differ in their reactivity and damaging effects in human aortic endothelial cells (HAECs) due to their distinct chemical structures [11, 12]. However, to our knowledge, no study has identified the protein targets of CRA-induced damage in HAECs or the mechanism underlying CRA cytotoxicity-induced protein damage; therefore, the molecular mechanisms underlying VI development remain unclear. Moreover, simultaneous bulk detection of proteins is difficult due to the limitations posed by traditional methods, which are often labor-intensive and costly. However, proteomics has enabled the systematic characterization of the molecular mechanism of VI development and to explore new indicators and signaling pathways related to CRA exposure. This research aimed to identify differentially expressed proteins that could act as indicators of CRA exposure using proteomics and then to use these indicators to explore key therapeutic targets, such as DNA interstrand crosslinks (ICLs), or identify new signaling pathways induced by CRA using tandem mass tag (TMT)-based liquid chromatography-mass spectrometry (LC–MS/MS). Our results provide new insights into the mechanisms and pathogenesis of VI and suggest new treatment strategies.

## Materials and methods

### Materials

CRA (98%) was purchased from Shanghai Acmec Biochemical Co., Ltd. (123-73-9, Shanghai, China), and HAECs were purchased from BeNa Culture Collection (BNCC337727; Beijing, China). Fanconi anemia complementation group A (FANCA) small interfering RNA (siRNA) and siRNA-Mate were synthesized and provided by GenePharma Co., Ltd. (G04002, Shanghai, China). Glutathione peroxidase (GSH-Px) assay kits were obtained from the Jiancheng Bioengineering Institute (A005; Nanjing, Jiangsu, China). Phospho-extracellular signal-regulated kinase (P-ERK, ab176660), phospho-c-Jun N-terminal kinase (P-JNK, ab176645), phospho-p38 kinase (ab221013), cyclooxygenase-2 (COX-2, ab267646), Wnt3a (ab54479), β-catenin (ab16051), phospho- ErbB2 (ab101229), and phospho-ErbB4 (ab61059) ELISA kits were purchased from Abcam (Cambridge, UK), and mitochondrial membrane potential assay kits were obtained from Abbkine Scientific Co., Ltd. (KTA4001; Wuhan, Hubei, China).

### Knockdown of FANCA expression by siRNA

The FANC pathway is a major pathway involved in the repair of ICLs. ICLs generated by aldehydes are chemically unstable and are relatively easily reversed [13, 14]. Thus, there is no reliable quantitative method for ICLs generated by aldehydes in vivo. Therefore, ICLs were measured using FANC-deficient cells [15]. HAECs were cultured in DMEM (12100061, Thermo Fisher, Shanghai, China) with 10% inactivated FBS (80230-6412, Zhejiang Tianhang Biotechnology Co. Ltd., Zhejiang, China) in a 5% CO_2_ environment at 37 °C. siRNA-liposome complexes were prepared according to the manufacturer’s instructions and incubated at 25 °C for 10 min. Cultured HAECs were subsequently incubated with siRNA-liposome complexes for 8 h, and afterwards fresh DMEM with 10% inactivated FBS was prepared, and incubation was continued for an additional duration of 48–96 h at 37 °C in a 5% CO_2_ environment.

### HAEC survival assays

HAECs were prepared as per methods described in “Knockdown of FANCA expression by siRNA” section and then cultured in fresh medium for a further duration of 9‒12 h. The cells were subsequently maintained in a fresh medium to which different concentrations of CRA—0, 0.5, 1.0, 2.0, 3.0, and 4.0 µmol/L, with adjustments performed every 24 h—were added daily. The HAECs were rinsed twice with PBS after culturing for 8 days for the formation of colonies, and the number of colonies (≥ 50 cells per colony) was evaluated. Lethal dose (LD_50_) values were determined graphically at the point of 50% cell damage based on the survival curves generated.

### Detection of mitochondrial membrane potential

Mitochondrial membrane potential was analyzed as per the manufacturer’s instructions. Briefly, 100 μL/well carbocyanine dye JC-1 was added to a 96-well plate after treatment with CRA (LD_50_ = 0.71 μM) and incubated at 37 °C under light protection conditions for 10 min; the control was not exposed to CRA. An F-2500 Fluorescence Spectrophotometer, with λex of 529 nm and λem of 590 nm (251-0090, Hitachi High-Technologies Corporation, Tokyo, Japan), was used to determine the results.

### Measurement of GSH levels

Oxidative stress was confirmed by measuring intracellular GSH levels immediately after exposure to CRA (LD_50_ = 0.71 μM), as well as in the non-exposed control, using a GSH detection kit (A006-1-1, Jiancheng Bioengineering Institute, Nanjing, Jiangsu, China) as per the manufacturer’s instructions. Briefly, HAECs (1 × 10^6^) were mixed with 1 mM GSH and reagent 1 after CRA exposure, and incubated for 5 min at 37 °C. Thereafter, a mixture of reagents 2–5 was added to the samples and the mixture was incubated at 37 °C for 15 min, before results were detected using an absorbance microplate reader at a wavelength of 420 nm (SpectraMax^®^ iD3, Molecular Devices, LLC., San Jose, CA, USA).

### Analysis of mitogen-activated protein kinase pathways and Wnt3a, β-catenin, phospho-ErbB2, phospho-ErbB4, and COX-2 levels

The mitogen-activated protein kinase (MAPK) pathways and Wnt3a, β-catenin, phospho- ErbB2, phospho-ErbB4, and COX-2 levels were detected after exposure to CRA (LD_50_ = 0.71 μM) and in the non-exposed control, using ELISA kits (ab61059, ab101229, ab221013, ab267646; Cambridge, UK), simplestep ELISA kits (ab176660, ab176645; Cambridge, UK), or sandwich ELISA kits (ab54479 and ab16051; Cambridge, UK) following the manufacturer’s instructions.

Wnt3a and β-catenin levels were detected using sandwich ELISA. For this, 100 µL of each appropriately diluted sample was added to each well and incubated for 90 min at 37 °C. Next, the wells were washed twice with 200 μL PBS, and then 100 μL of the diluted detection antibody solution was added to each well. Finally, the plates were covered with an adhesive plastic and incubated for 2 h at 15–30 ℃. After incubation, the wells were washed four times with PBS; then, 100 μL of an HRP-conjugated secondary antibody was added to each well, and the plates were covered with an adhesive plastic and incubated for 1–2 h at 15–30 ℃.

P-ERK and P-JNK levels were detected using SimpleStep ELISA. Briefly, 50 µL of sample or control was added to appropriate wells, followed by the addition of 50 μL of an antibody cocktail and incubation for 1 h at 15–30 °C on a shaker set to 400 rpm. Each well was then washed three times with 350 μL of wash buffer.

P-P38, P-ErbB2, P-ErbB4, and COX-2 levels were measured using ELISA kits. In brief, 100 µL of sample or standard was added to appropriate wells and incubated for 2.5 h at 15–30 °C. Sample wells were then washed four times with a wash solution, followed by the addition of 100 μL of a biotinylated antibody, covering of the plates, and incubation for 1 h at 15–30 ℃. The wells were washed as described previously; then, 100 μL of the prepared streptavidin solution was added to each well, and the plates were again covered and incubated for 45 min at 15–30 ℃.

Finally, for all ELISAs, wells were washed as before with a wash buffer; 100 μL of the TMB substrate was added to each well, and the plates were covered and incubated for 15–30 min in the dark on a shaker set to 400 rpm. Then, 100 μL of a stop solution was added to each well, and the plates were recovered and placed on a shaker for 1 min to mix the contents. The optical density (OD450nm) of each well was measured at 450 nm.

### TMT-based proteomics analysis

#### Protein preparation and TMT labeling

Protein extraction was performed as follows: all samples were homogenized in lysis buffer (4% SDS, 1 mM DTT, 150 mM Tris–HCl pH 8.0, protease inhibitor) and incubated for 5 min in boiling water, and the homogenate was sonicated on ice. The crude extract was then incubated in boiling water again and centrifuged at 16,000×*g* at 25 °C for 10 min. Protein content was determined, and the supernatants were stored at − 80 °C until use. Briefly, 200 μg of protein, from each sample, was added to 30 µL SDT buffer (4% SDS, 100 mM DTT, and 150 mM Tris–HCl; pH 8.0). The detergent, DTT, and other low-molecular-weight components were removed using UA buffer (8 M Urea and 150 mM Tris–HCl; pH 8.0) via repeated ultrafiltration (Pall units, 10 kD). Next, 100 μL of 0.05 M iodoacetamide in UA buffer was added to block reduced cysteine residues, and the samples were incubated for 20 min in darkness. The filters were washed three times with 100 μL UA buffer, and then with 100 μL DS buffer (50 mM triethylammoniumbicarbonate at pH 8.5) twice. Finally, the protein suspensions were digested with 2 μg trypsin (Promega) in 40 μL DS buffer overnight at 37 °C, and the peptides formed were collected as a filtrate [16] and finally vacuum dried.

#### Peptide fractionation with strong cation exchange chromatography

Strong cation exchange chromatography was briefly performed using the AKTA Purifier system (GE Healthcare, Illinois, USA). Gradient elution involving 0–10%, 10–20%, 20–45%, and 50–100% of buffer B (500 mM KCl and 10 mM KH2PO4 in 25% acetonitrile; pH 2.7) was used to elute the peptides at a flow rate of 1 mL /min for 2, 25, 5, and 5 min, respectively; peptides were collected according to the manufacturer’s instructions. All fractions were pooled and desalted with C18 standard density cartridges (Empore SPE, 7 mm/3 mL; Sigma-Aldrich, Missouri, USA), followed by vacuum centrifugation and reconstitution of the peptides with trifluoroacetic acid.

#### Liquid chromatography-electrospray ionization MS/MS analysis

We used a Q Exactive HF-X orbitrap mass spectrometer coupled with Easy nLC (Thermo Fisher Scientific, MA, USA). Briefly, 5 μL of each fraction was injected for nanoLC-MS/MS analysis. The peptide mixture was then loaded onto a C18-reversed phase column (15 cm long, 75 μm inner diameter) packed in-house with RP-C18 5 μm resin in a buffer (0.1% Formic acid) and separated with a linear gradient of buffer (80% acetonitrile and 0.1% Formic acid) at a flow rate of 250 nL/min controlled using IntelliFlow technology over 90 min. MS data were then acquired using a data-dependent top20 method, dynamically choosing the most abundant precursor ions from the survey scan (300–1800 m/z) for HCD fragmentation. Determination of the target value was based on predictive Automatic Gain Control (pAGC). Dynamic exclusion duration was 60 s.

#### Sequence database searching and data analysis

The samples were analyzed using Mascot 2.2 (Matrix Science, London, UK), Proteome Discoverer 1.4 (ThermoFisher Scientific), the UniProt Mus musculus database, with 87,874 sequences downloaded on 3/26/2020, and a decoy database.

Parameters against which samples were measured included the following: peptide mass tolerance, 20 ppm; MS/MS tolerance, 0.1 Da; enzyme, trypsin; missed cleavage, 2; fixed modification: carbamidomethyl (C), TMT16 (K), TMT16 (N-term); variable modification: oxidation (M); false discovery rate ≤ 0.01.

#### Bioinformatics

The Blast2GO bioinformatics platform (https://www.blast2go.com/) was used to determine Gene Ontology (GO) annotated histograms. Protein pathways were analyzed using the Kyoto Encyclopedia of Genes and Genomes (KEGG) database on the KEGG Automatic Annotation Server (KAAS) program (http://www.genome.jp/kaas-bin/kaas_main). STRING (http://string-db.org/) was used to determine protein–protein interaction (PPI) networks. Four data sources—genomic context, high-throughput experiments, coexpression, and previous knowledge—were used to evaluate direct or indirect PPIs according to STRING.

### Parallel reaction monitoring

Proteins related to diagnostic and therapeutic targets were confirmed using parallel reaction monitoring (PRM) analysis. The target peptide was prepared as per methods described in “Protein preparation and TMT labeling” section, and the label-free protocol was used to prepare 72 peptides. The stable isotope AQUA peptide was used to prepare the standard internal reference. Tryptic peptides were loaded onto the stage tips of C18 columns for desalting prior to reversed-phase chromatography on an nLC-1200 Easy system (Thermo Scientific). Subsequently, liquid chromatography was performed with 5–35% acetonitrile gradients for 45 min, and Q Exactive Plus MS was used for conducting PRM analysis. An optimized protocol was used to process data on the energy of collision, state of charge, and retention time of the target peptides, including unique peptides with high intensities. The Skyline application (MacCoss Lab Software, Seattle, WA, USA) was used to analyze raw data, in which the intensity of the signal produced by a certain peptide sequence can be quantified according to each sample and referenced to standards via normalization of each protein.

### Statistical analysis

MS data were analyzed using the MaxQuant software version 1.6.5.0 (http://maxquant.org/). Functional annotations were performed using the Database for Annotation, and PPI analysis was performed using the STRING database. Data are presented as mean ± standard deviation, based on the results of three to five repetitions. The Student’s *t*-test was used to evaluate the statistical differences for paired data, and statistical analyses were performed using the SPSS software version 22.0 (IBM Corp., Armonk, NY, USA). Values of *p* < 0.05 were considered statistically significant.

## Results

### Partial inactivation of HAECs by CRA confirmed through DNA damage, disrupted GSH, depleted mitochondrial membrane potential, as well as MAPK pathway activation and COX-2 expression

**Fig. 1:**
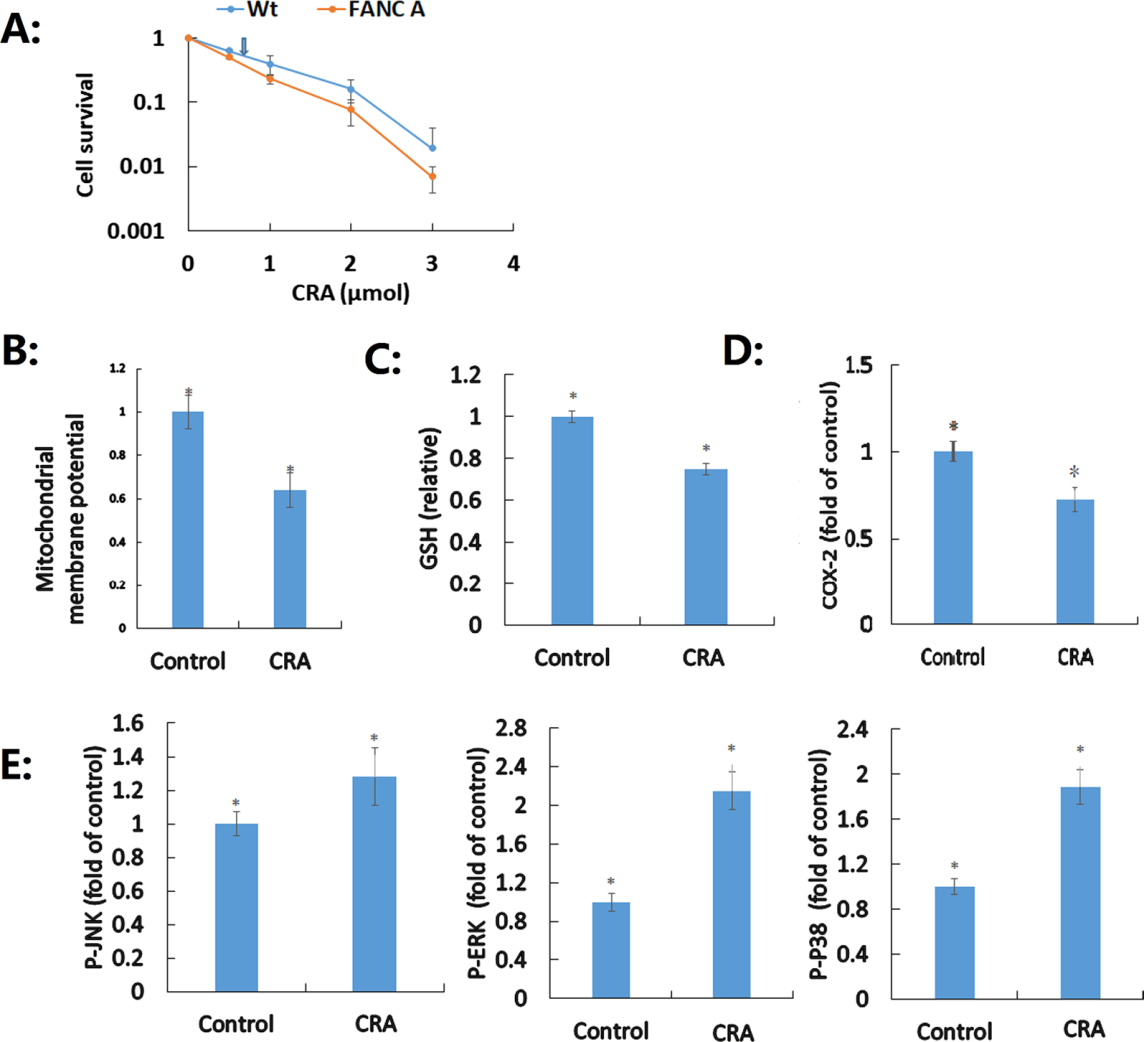
The effects of crotonaldehyde (CRA) on human aortic endothelial cells (HAECs). **A** DNA damage induced by CRA in HAECs deficient in the FANC pathway. HAECs were exposed to a range of CRA concentrations—0, 0.5, 1, 2, 3, and 4 μM—and incubated for a period of 8 days. Drug lethal dose (LD_50_) values were identified from data generated using survival curves and have been indicated by an arrow. Data are expressed as mean ± standard deviation of five independent experiments. **B** Effect of CRA on the depletion of mitochondrial membrane potential. **C** Effect of CRA on GSH levels. **D** Activation of COX-2 by CRA. **E** Activation of MAPK pathways, including P-ERK, P-JNK, and P-P38, by CRA. Data are expressed as mean ± standard deviation of three independent experiments (**B**–**E**); statistically significant differences are indicated by an asterisk; *p* < 0.05 was considered statistically significant

HAECs were exposed to CRA to evaluate its effect on inactivated HAECs; the control was not exposed to CRA. To examine the differentially expressed proteins that could serve as potential indicators, we used the proteomics approach. Based on the identified novel indicators, we explored the related key therapeutic targets, such as ICLs (Additional file shows this in more detail—see Additional files 1, 2, 3, 4, 5 and 6). DNA damage was determined as one of the mechanisms by which CRA induced VI according to indicators, as evidenced by the CRA-resistance of HAECs (LD_50_ = 0.71 μM) compared with HAECs deficient in the FANC pathway (LD_50_ = 0.48 μM). ICLs are one of the most significant DNA damages, the accumulation of which results in growth arrest and cell death; the FANC pathway is capable of rescuing ICL-stalled replication forks while maintaining the genetic stability of daughter cells to ensure survival. This study clarified the important role of ICLs, but not DNA double strand breaks and DNA–protein crosslinks, and the involvement of the FANC pathway in the removal of ICLs induced by CRA (Fig. 1A).

Six differentially expressed proteins (Table 1) as indicators suggested that CRA-induced mitochondrial respiratory chain increases the production of reactive oxygen species and perturbs GSH, resulting in oxidative stress. The levels of mitochondrial membrane potential (64% relative to the negative control group; Fig. 1B) and GSH (75% relative to the negative control group; Fig. 1C), which are indicative of oxidative stress, were confirmed after CRA exposure (LD_50_ = 0.71 μM).

**Table 1:** The indicators of vascular injury

GO analysis	Protein accession	Protein	Gene	FC^1^	FC^2^	Node
DNA damage and repair	Q5TG38	DNA methyltransferase 1-associated protein 1 (Fragment)	DMAP1	0.52	0.45	1
	Q9BY89	Uncharacterized protein KIAA1671	KIAA1671	2.54	2.36	0
	D3DXI9	DNA polymerase epsilon catalytic subunit	POLE	1.86	1.69	0
	F8WBH5	Proteasome activator complex subunit 4	PSME4	2.16	1.99	0
	P01023	Alpha-2-macroglobulin	A2M	1.80	2.04	4
	Q8TC04	Keratin 23 (Histone deacetylase inducible)	KRT23	0.65	0.59	1
Oxidation–reduction process	H0YL12	Electron transfer flavoprotein subunit alpha, mitochondrial (Fragment)	ETFA	2.03	1.87	3
	O95101	Cytochrome c oxidase subunit	N/A	0.56	0.51	0
	B4DWB3	cDNA FLJ61432, highly similar to Transient receptor potential cation channel subfamily M member 2		0.49	0.44	0
Mitochondrial	P48507	Glutamate–cysteine ligase regulatory subunit	GCLM	1.88	2.04	1
	E7EVY0	Mitochondrial inner membrane protein OXA1L	OXA1L	1.82	1.76	4
	H0YEL3	V-type proton ATPase subunit a (Fragment)	TCIRG1	0.00	0.14	0
Arterial dysfunction	B4DFV1	Protein kinase C (EC 2.7.11.13)		0.45	0.31	0
	P48507^a^	Glutamate–cysteine ligase regulatory subunit (GCS light chain) (Gamma-ECS regulatory subunit) (Gamma-glutamylcysteine synthetase regulatory subunit) (Glutamate–cysteine ligase modifier subunit)	GCLM GLCLR	1.88	2.04	1
Vascular remodeling	B4DI57	cDNA FLJ54111, highly similar to Serotransferrin	N/A	2.32	1.96	0
	B4DV58	cDNA FLJ55250, highly similar to Cytochrome P450 4B1	N/A	1.52	1.72	0
	C9J080	Beta-adducin (Fragment)	ADD2	0.00	0.11	0
	P05109	Protein S100-A8	S100A8	0.66	0.57	1
	Q8TC04^a^	Keratin 23 (Histone deacetylase inducible)	KRT23	0.65	0.59	1
	A0A1B0GV06	Acid ceramidase (Fragment)	ASAH1	0.00	0.05	1
Un-integrity and dysfunction of HAECs	P48507^a^	Glutamate–cysteine ligase regulatory subunit (GCS light chain) (Gamma-ECS regulatory subunit) (Gamma-glutamylcysteine synthetase regulatory subunit) (Glutamate–cysteine ligase modifier subunit)	GCLM	1.88	2.04	1
	A0A0X1KG69	InaD-like protein	PATJ	3.27	2.79	0
	E9PP49	Collagen alpha-2(VIII) chain (Collagen type VIII alpha 2)	COL8A2	2.21	2.54	0
Lipid metabolism	P19838	Nuclear factor NF-kappa-B p105 subunit (DNA-binding factor KBF1) (EBP-1) (Nuclear factor of kappa light polypeptide gene enhancer in B-cells 1) [Cleaved into: Nuclear factor NF-kappa-B p50 subunit]	NFKB1	0.42	0.55	0
	A0A2P9AF66	3′(2′),5′-bisphosphate nucleotidase CysQ	cysQ	1.75	2.01	0
	B4DJ23	Myotubularin-related protein 14 (cDNA FLJ52857)	MTMR14	0.16	0.12	0
	A0A2P9AJA0	Histidine kinase	BQ8482_180436	2.78	3.25	0
Immunity and autophagy	P19838^a^	Nuclear factor NF-kappa-B p105 subunit (DNA-binding factor KBF1) (EBP-1) (Nuclear factor of kappa light polypeptide gene enhancer in B-cells 1) [Cleaved into: Nuclear factor NF-kappa-B p50 subunit]	NFKB1	0.42	0.55	0
	P05109^a^	Protein S100-A8	S100A8	0.66	0.57	1
	P05546	Heparin cofactor 2	SERPIND1 HCF2	2.28	2.05	1
	B4DJ23^a^	Myotubularin-related protein 14 (cDNA FLJ52857)	MTMR14	0.16	0.12	0
	P02765	Alpha-2-HS-glycoprotein	AHSG	2.19	1.96	7
MAPK cascade	B4DI57^a^	cDNA FLJ54111, highly similar to Serotransferrin	N/A	2.32	1.96	0
	P19838^a^	Nuclear factor NF-kappa-B p105 subunit (DNA-binding factor KBF1) (EBP-1) (Nuclear factor of kappa light polypeptide gene enhancer in B-cells 1)	NFKB1	0.42	0.55	0
	Q9UPS8	Ankyrin repeat domain-containing protein 26	ANKRD26	1.68	1.73	1
Blood coagulation	B4DPN0	cDNA FLJ51265, moderately similar to Beta-2-glycoprotein 1 (Beta-2-glycoprotein I)	N/A	1.56	1.68	0
	P05546^a^	Heparin cofactor 2	SERPIND1 HCF2	2.28	2.05	1
	P02765^a^	Alpha-2-HS-glycoprotein	AHSG	2.19	1.96	7
	Q13797	Integrin alpha-9 (Integrin alpha-RLC)	ITGA9	1.97	2.14	0
ERBB	B4DFV1^a^	Protein kinase C (EC 2.7.11.13)	N/A	0.45	0.31	0
	Q96CD4	Similar to breast cancer anti-estrogen resistance 1	N/A	1.65	1.51	0
	Q9Y4L5	E3 ubiquitin-protein ligase RNF115	RNF115	1.82	2.21	3
Wnt signaling pathway	A0A024R2V4	Coiled-coil domain containing 51, isoform CRA_a	CCDC51	1.67	1.59	0
	P19838^a^	Nuclear factor NF-kappa-B p105 subunit (DNA-binding factor KBF1) (EBP-1) (Nuclear factor of kappa light polypeptide gene enhancer in B-cells 1) [Cleaved into: Nuclear factor NF-kappa-B p50 subunit]	NFKB1	0.42	0.55	0
	Q8N7H5	RNA polymerase II-associated factor 1 homolog (hPAF1) (Pancreatic differentiation protein 2)	PAF1 PD2	0.66	0.53	0
	B4DI57^a^	cDNA FLJ54111, highly similar to Serotransferrin	N/A	2.32	1.96	0
	Q4ZG84	Submitted name: Uncharacterized protein LRP2	LRP2	1.55	1.86	6

*FC*^*1*^ determination the FC with TMT-based proteomics analysis; *FC*^*2*^ validation of FC1 by PRM method

^a^This protein occurs more than once

Moreover, five differentially expressed proteins (Table 1) as indicators suggested that immunity was closely associated with VI after exposure to CRA through inhibition of COX-2 (72.3%, Fig. 1D). Three indicators (Table 1) were closely associated with activation levels of MAPK pathways (Fig. 1E), and thus these pathways were confirmed to have the potential to be applied as biomarkers or therapeutic targets after CRA exposure. Compared with those in the negative control group, inhibition levels of COX-2 and activation levels of MAPK pathways, which are related to vascular remodeling, disintegrity, and dysfunction of HAECs as well as inflammation increased after CRA exposure.

### Confirmation of the activation of Wnt and ErbB signaling pathways by CRA based on Wnt3A, β-catenin, Phosphor-ErbB2, and Phospho-ErbB4 protein levels, respectively

**Fig. 2:**
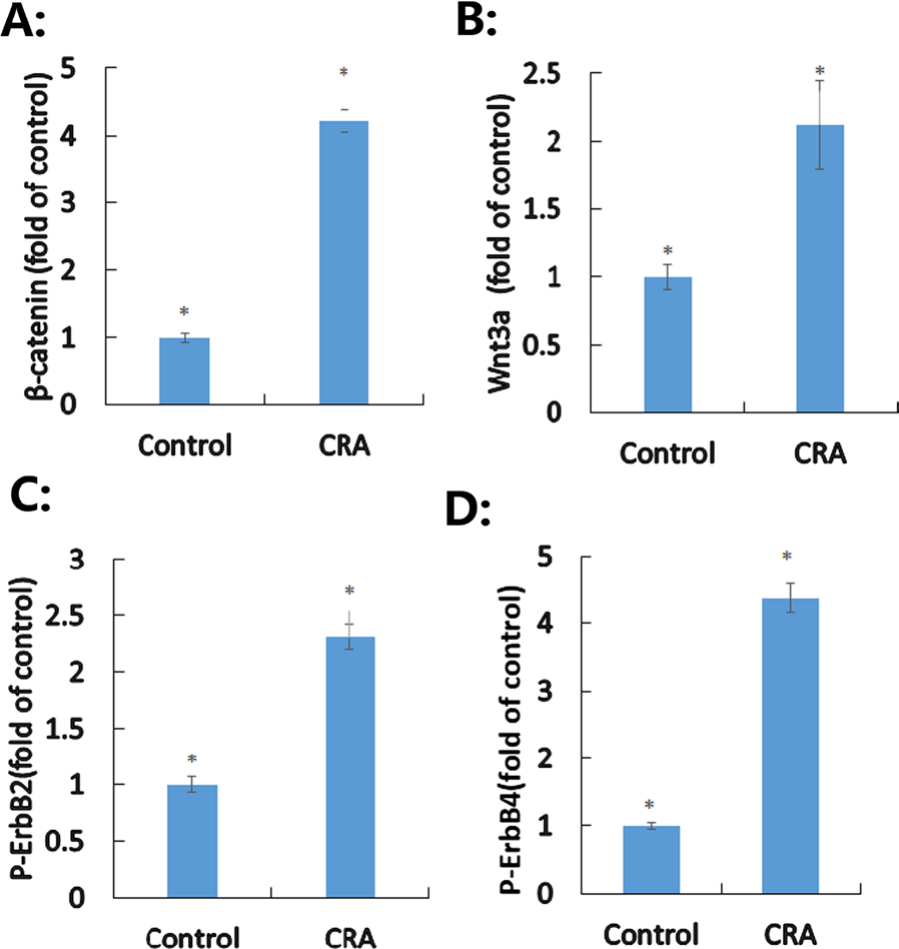
Effects of crotonaldehyde (CRA) on the Wnt and ErbB signaling pathways. **A** Activation of β-catenin by CRA. **B** Activation of Wnt3a by CRA. **C** Activation of phospho-ErbB2 by CRA. **D** Activation of phospho-ErbB4 by CRA. *p < 0.05

Five differentially expressed proteins associated with the Wnt signaling pathway and three associated with the ErbB signaling pathway were used as new indicators. Activation of the Wnt signaling pathway was determined as one of the primary mechanisms underlaying VI pathogenesis, as evidenced by the levels of both β-catenin (422%, Figure 2A) and Wnt3a (212%, Figure 2B), which regulate processes involved in vascular remodeling [17]. Significant increases in the levels of phospho- ErbB2 (231%, Figure 2C) and phospho-ErbB4 (437%, Figure 2D), relative to those in the negative control group, which are indicative of ErbB signaling pathway activation, were observed following CRA exposure (LD_50_ = 0.71 μM).

### Quantitative proteomic analysis of HAECs

Following CRA exposure (LD_50_ = 0.71 μM), a total of 3797 proteins and 14,218 peptides were determined using LC–MS/MS (Additional file shows this in more detail—see Additional files 1, 2, and 3) compared to those in the negative control. With reference to expression levels determined in the negative control group, 68 upregulated proteins and 87 downregulated were found according to fold change (FC) cutoff values (FC > 1.5 or < 0.667) (Additional file shows this in more detail—see Additional file 4). Protein expression patterns were significantly different between the exposed and negative control groups (Additional file shows this in more detail—see Additional file 7).

### Differentially expressed proteins analyzed through KEGG pathway annotation and enrichment

**Fig. 3:**
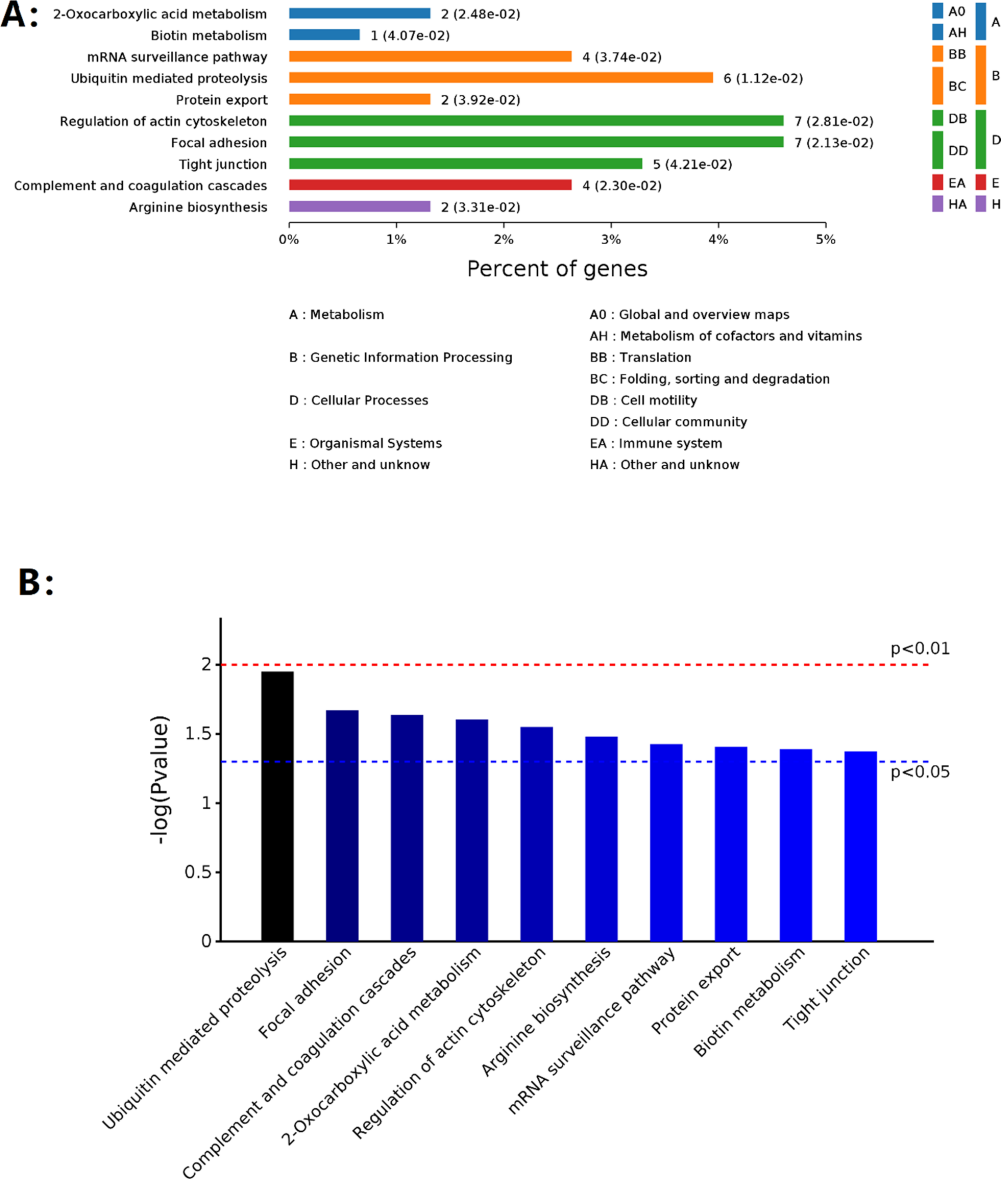
Differentially expressed proteins determined using Kyoto Encyclopedia of Genes and Genomes (KEGG) pathway annotations. **A** The abscissa represents the percentage of genes; the ordinate lists the KEGG pathways. **B** The blue and red lines indicate *p* < 0.05, and *p* < 0.01, respectively

A total of 155 differentially expressed proteins were analyzed using KEGG pathway enrichment analysis. The most important annotated KEGG pathways were cellular processes that involved regulation of the actin cytoskeleton, focal adhesion, and tight junctions. This highlights the direct relation of CRA exposure to vascular remodeling, disintegrity, and dysfunction of HAECs as well as inflammation. Furthermore, data on genetic information processing, such as ubiquitin-mediated proteolysis and mRNA surveillance pathways, suggested that CRA exerted a strong influence on DNA damage and repair. Finally, complement and coagulation cascades indicated that CRA affected blood clotting and inflammation (Fig. 3). More importantly, we provided unique insights into new disease pathways involving the Wnt and ErbB signaling pathways (Table 1 and additional file show this in more detail—see Additional files 4 and 5).

### GO annotation enrichment analysis

**Fig. 4:**
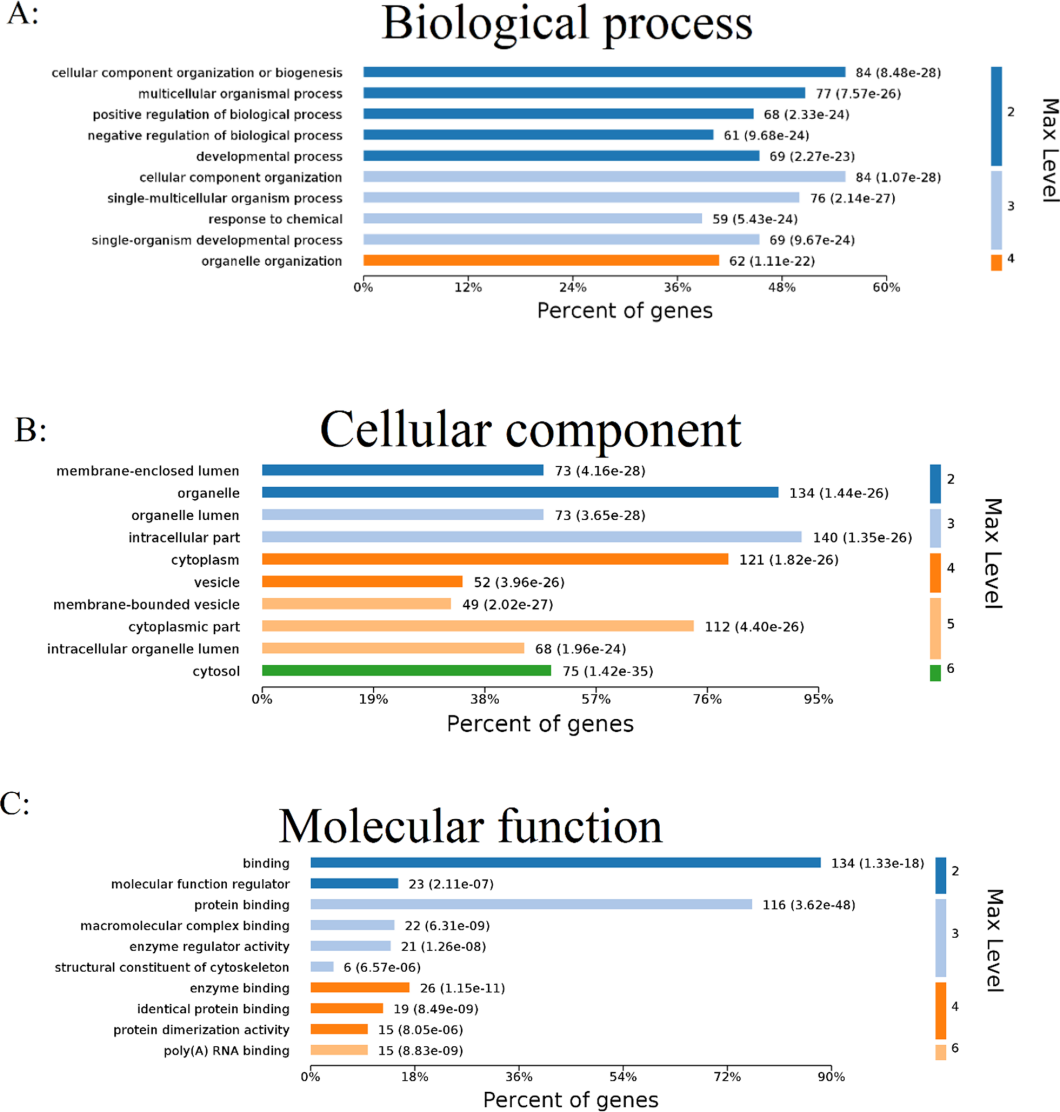
Expression of differentially expressed proteins was analyzed using Gene Ontology (GO) enrichment analysis. The top ten biological process (**A**), molecular function (**B**), and cellular component (**C**) enrichment terms are shown. The abscissa represents the percentage of genes, while the ordinate indicates the details of the enrichment terms (*p* < 0.01)

Cellular components, molecular functions, and biological processes were evaluated during GO analysis. The GO terms “cellular component organization or biogenesis” and “positive/negative regulation of biological process” exhibited the highest percentages (Fig. 4A). Cellular component GO analysis indicated that the “intracellular part,” “organelle” such as mitochondria, and “cytoplasm” were the most important factors (Fig. 4B). According to molecular function GO analysis, terms such as “binding” or “protein binding” were highly enriched, indicating that CRA exerted a robust influence on protein levels (Fig. 4C). Furthermore, we analyzed the levels of all differentially expressed proteins and investigated the Wnt and ErbB signaling pathways (Table 1 and additional file show this in more detail—see Additional files 4 and 5) to expand our understanding of the outcomes of GO annotation enrichment analysis of the profiles of molecular mechanisms, offering new insights into the mechanism of VIs induced by CRA (Table 1).

### PPI Network for the differentially expressed proteins

**Fig. 5:**
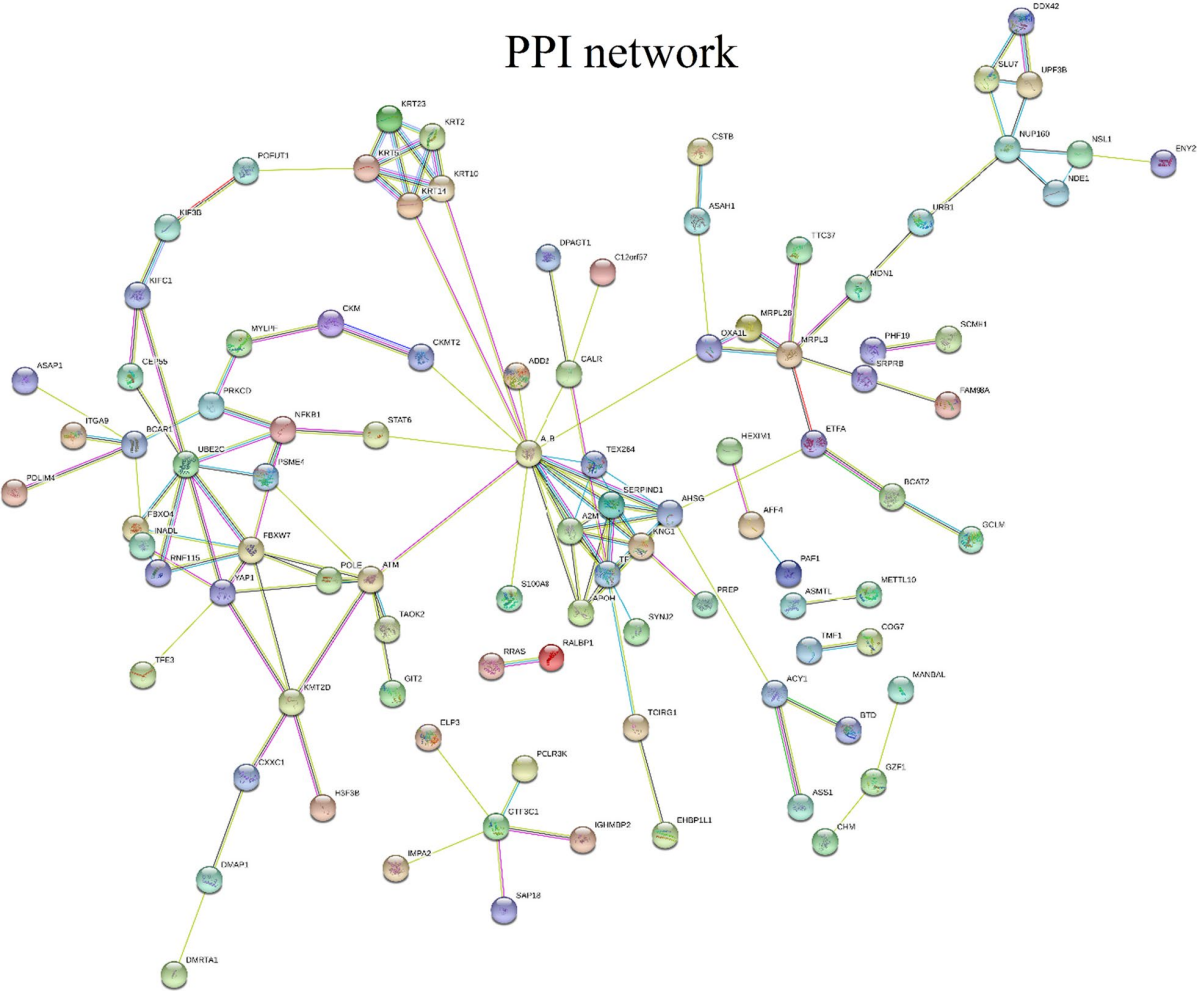
Protein–protein interactions between the differentially expressed proteins were determined using the STRING database. Nodes represent the proteins, and lines represent protein–protein interactions

The STRING interaction scores (Fig. 5) were used to construct a PPI network to broaden our understanding of the mechanism of VI induced by CRA. Protein interaction pairs were sorted into related functional groups to reveal protein-to-protein networks and “communities,” and the number of nodes connected to any given node was evaluated (Table1). The key factors affecting the PPI network were proteins with high connectivity or high FC.

### Validation of proteins by PRM

Based on the outcomes of GO annotation enrichment and KEGG pathway annotation and enrichment analyses, we manually analyzed all differentially expressed proteins (the number of peptides used to identify a protein can be obtained using the field “#Peptides” (see Additional files 2 and 3), and the information on the sequence and charge state of each peptide can be obtained using the field “Sequence” (see Additional file 3), determining certain key points as indicators in Table 1. Protein expression levels were confirmed using PRM analysis (Table 1).

## Discussion

CRA is a short α, β-unsaturated aldehyde, one of the top nine pollutants from cigarette smoke and e-cigarette vapors—the major non-occupational sources [18]—and is generated outdoors via air pollution arising from vehicle exhaust fumes and industrial emissions, amongst others [19]. Cardiovascular disease-related mortality arising from environmental pollution exceeds cancer-related mortality rates [20], and CRA has been identified as a major factor closely related to the development of VI [7, 8, 10].

Aldehydes, including simple aldehydes (formaldehyde), short α, β-unsaturated aldehydes (CRA and others), or long α, β-unsaturated aldehydes (2,4-decadienal), differ in their reactivity and damaging effects on HAECs due to their different chemical structures [11, 12]. However, to the best of our knowledge, no study has established the critical targets or elucidated any novel molecular mechanisms underlying CRA cytotoxicity in HAECs. This study offers new insights into these aspects. First, we overcame the challenges of conventional technology, such as simultaneous detection of substantial numbers of proteins, provided clarification on relevant molecular profiles, and performed manual classification of all differentially expressed proteins (Additional file shows this in more detail—see Additional files 4, 5, and 6) as new indicators. The indicators covered all aspects of VI and provided a basis for further studies on the mechanism of VI induced by CRA. The experiments performed herein to confirm newer ideas on VI include those on DNA damage (Fig. 1A), oxidative stress and mitochondrial dysfunction (Fig. 1B, C), inflammation (Fig. 1D), arterial dysfunction and structural changes in the vasculature (Fig. 1E), and autophagy (Fig. 1A–E), using TMT-based LC–MS/MS and bioinformatics analysis. Second, according to the identified indicators, this study is the first to elucidate the underlying mechanisms and possible therapeutic strategies, including the Wnt and ErbB signaling pathways, for VI induced by CRA. Finally, the identified indicators can be used as clues to guide further clinical studies to explore the relationship between the differentially expressed proteins and VI induced by CRA.

The upregulated (FC = 2.54) and uncharacterized protein KIAA1671 (protein accession number Q9BY89) interacts with nucleolar proteins to maintain DNA stability [21]. Conversely, the expression of the Dmap1 gene (protein accession number Q5TG38), which is responsible for several important functions in the DNA repair process for maintenance of DNA stability [22], was downregulated (FC = 0.52), which implied that CRA exposure weakened DNA repair. The catalytic subunit of the upregulated (FC = 1.86) DNA polymerase epsilon (protein accession number D3DXI9) acts as the major leading-strand DNA polymerase to repair DNA damage [23], whereas the upregulation (FC = 1.80) of alpha-2-macroglobulin (protein accession number P01023) is related to inhibition of DNA fragmentation, which is a hallmark of CRA-induced apoptosis [24]. Upregulation (FC = 2.16) of proteasome activator complex subunit 4 (protein accession number F8WBH5) is related to signal transduction by p53 class mediator, resulting in cell cycle arrest to repair DNA damage [25], whereas downregulation (FC = 0.65) of keratin 23 (protein accession number Q8TC04) implies that CRA exposure weakened the expression of genes involved in DNA damage response, mainly molecules of the double strand break repair and homologous recombination pathways [26]. These new indicators among the differentially expressed proteins are closely associated with the DNA repair process (Table 1). Although previous research has focused on DNA damage, the most important types of DNA damage in CRA-induced VI remain unclear [4, 6, 27]. This research confirmed that ICLs are amongst the most serious types of DNA damages induced by CRA, which result in growth arrest and cell death on accumulation. The FANC pathway is involved in the removal of ICLs induced by CRA (Fig. 1A), which is different from that observed with simple aldehydes (formaldehyde) or long α, β-unsaturated aldehydes (such as 2,4-decadienal). This is the first report to suggest ICLs and the FANC pathway as diagnostic biomarkers or therapeutic targets in CRA-induced DNA damage and repair.

V-type proton ATPase subunit a (protein accession number H0YEL3, FC = 0.00) was markedly damaged by CRA, although it is closely related to energy production promotion and confers health-promoting functions in porcine intestinal epithelial cells [28]. Upregulation (FC = 1.82) of the mitochondrial inner membrane protein OXA1L (protein accession number E7EVY0) effectively promotes the assembly of multiple respiratory chain complexes [29], while increasing the expression (FC = 1.88) of glutamate-cysteine ligase modifier (GCLM) (protein accession number P48507), which promotes the expression of GSH. The upregulation of these two proteins corresponds to the mitochondrial efforts to maintain their function. The differentially expressed proteins as indications suggested that CRA-induced mitochondrial respiratory chain increases the production of reactive oxygen species, resulting in oxidative stress, while producing ATP (Fig. 1B). This study also confirmed the damage of CRA to HAECs by perturbing GSH and mitochondrial membrane potential (Fig. 1B, C), which is different from that observed with simple aldehydes or long α, β-unsaturated aldehydes. Upregulation (FC = 2.03) of electron transfer flavoprotein subunit alpha, mitochondrial (fragment) (protein accession number H0YL12) may contribute to GSH-Px activity, conferring more effective protection against reactive oxygen [30]. Furthermore, downregulation (FC = 0.49) of cDNA FLJ61432 (highly similar to transient receptor potential cation channel, subfamily M, member 2; protein accession number B4DWB3) effectively inhibits oxidative stress induced by CRA [31]. Finally, cytochrome c oxidase (CcO) (protein accession number O95101) consists of 13 subunits and is the terminal complex of the mitochondrial electron transport chain, which is a key component of oxidative metabolism; decreased expression (FC = 0.56) of CcO results in CRA-induced oxidative stress.

Based on the close association between these indicators and MAPK pathways, activation of these pathways confirmed their potential applicability as biomarkers or therapeutic targets after CRA exposure (Fig. 1E). Downregulation of NFKB1 caused increased expression of both NF-κB and MAPK through loss of signaling repressors [32]. The expression (FC = 2.32) of serotransferrin (protein accession number B4DI57; cDNA FLJ54111, highly similar to serotransferrin) improves BMP-p38 MAPK signaling and increases the expression of the Wnt inhibitor sclerostin [33], whereas upregulation (FC = 1.68) of ankyrin repeat domain-containing protein 26 (protein accession number Q9UPS8) effectively activates the MAPK/ERK signaling pathway [34]. Previous studies have shown that P-JNK and P-ERK induce human cell apoptosis [35, 36].To the best of our knowledge, this is the first study to directly assess the relationship among HAEC apoptosis, arterial dysfunction, and structural changes in the vasculature and the activation of MAP kinase pathway induced by CRA.

Indicators related to arterial dysfunction were first identified by the downregulation (FC = 0.45) of protein kinase C (protein accession number B4DFV1), resulting in enhanced Ca^2+−^sensing receptor-mediated relaxation of phenylephrine-contracted mesenteric arteries [37]. Further, acute high shear stress caused significant upregulation of Nrf2 target genes, heme oxygenase-1 (HO-1) and GCLM, and increased expression of the latter might be deleterious to vascular cells in the context of acute hemodynamic injury [38].

The activation of MAPK pathways and occurrence of DNA damage, oxidative stress, and arterial dysfunction can directly induce structural changes in the vasculature. This study identified certain proteins as new indicators of VI. Owing to its downregulation, keratin 23 could not function effectively in constituting a reservoir protein necessary for the creation of a more robust cytoskeleton capable of withstanding shear forces [39]. Conversely, upregulation (FC = 1.52) of cDNA FLJ55250 (highly similar to cytochrome P450 4B1; protein accession number B4DV58) mediates vascularization [40]. Downregulation (FC = 0.66) of S100A8/A9 (protein accession number P05109), regulators of macrophage polarization downstream of vascular endothelial growth factor receptor 1, mediated vascular inflammation, vascular calcification, and vascular oxidative stress [41, 42]. We also investigated adducin, the heterodimeric cytoskeleton protein whose three subunits are encoded by ADD1, ADD2 (protein accession number C9J080, FC = 0.00), and ADD3, respectively; the role of adducin in hypertension and its downregulation-related disorders have been reported [43]. Previous research showed a close association between significantly higher (FC = 2.32) levels of transferrin receptor 1 (protein accession number B4DI57) and atheroma in male patients, whereas downregulation of acid ceramidase (protein accession number A0A1B0GV06, FC = 0.00) induces arterial medial calcification through enhanced small extracellular vesicle release [44]. These indicators strongly suggest that CRA is associated with arterial dysfunction and structural changes in the vasculature. One study confirmed that acute exposure to high concentrations of CRA induces arterial dysfunction and structural changes in the vasculature through activated MAPK pathways [45]. Meanwhile, we confirmed that the activation of MAPK pathways can directly induce structural changes and arterial dysfunction in the vasculature with low concentrations of CRA (Fig. 1E).

The differentially expressed proteins as indicators also suggested that immunity was closely associated with VI after exposure to CRA. Downregulation of NFKB1 further causes increased expression of TNF as well as signal transducer and activator of transcription (STAT), resulting in an inflammatory immune response [46]. Upregulation (FC = 2.19) of serum alpha-2-HS-glycoprotein (AHSG) (protein accession number P02765) effectively blocks transforming growth factor beta (TGF-β)1 from binding to cell surface receptors, suppresses TGF-β signal transduction, and inhibits macrophage activation [47], indicating that HAECs attempt to reduce inflammation damage after CRA exposure. During inflammation, S100A8 is actively released and plays a crucial role in modulating the inflammatory response by stimulating leukocyte recruitment and inducing cytokine secretion [48]. An antimicrobial helix A-derived peptide of heparin cofactor II (protein accession number P05546; FC = 2.28) blocks endotoxin responses in vivo, suppresses pro-inflammatory cytokines, reduces vascular leakage and infiltration in lung tissue, and normalizes coagulation to reduce inflammation damage after CRA exposure [49]. The downregulation of COX-2 expression levels (Fig. 1D) confirmed that an immune response was triggered after exposure to CRA. These results are in line with previous studies, though they focused on the treatment of neuroinflammatory diseases, such as Alzheimer's disease [50] or anti-arthritic therapy [51].

Blood coagulation dysfunction was further induced by CRA exposure. Upregulation (FC = 2.28) of heparin cofactor 2 (protein accession number P05546) effectively inactivates thrombin in the presence of dermatan sulfate [52]. It has also been suggested that AHSG plays an indirect role in the pathogenesis of ischemic stroke through its influence on stroke risk factors, while also exhibiting association with the development of cardiovascular disease [53]. Beta-2-glycoprotein I (protein accession number B4DPN0, FC = 1.56) can upregulate and downregulate coagulation systems based on external conditions [54], whereas integrin alpha-9 (protein accession number Q13797; FC = 1.97) mediates adhesion and migration to thrombin-cleaved osteopontin [55].

Five differentially expressed proteins related to the Wnt signaling pathway were used as new indicators (Table 1 and additional file show this in more detail—see Additional files 5 and 6). The expression (FC = 1.67) of coiled-coil domain containing 51, isoform CRA_a (protein accession number A0A024R2V4) forms the structural basis for auto-inhibition of the Wnt pathway through homomerization of the DIX domain [56]. NFKB1 is involved in protein polyubiquitination and the Wnt signaling pathway [57]. The PAF1 complex, involved in the expression (FC = 0.66) of RNA polymerase II-associated factor 1 homolog (protein accession number Q8N7H5), establishes interaction with histone-modifying enzymes and RNA polymerase II to regulate transcription, which is required for nuclear transduction of the Wnt signal, and binds directly to the C-terminal region of beta-catenin/Armadillo [58]. Serotransferrin (protein accession number B4DI57, cDNA FLJ54111, highly similar to serotransferrin; FC = 2.32) improved BMP-p38MAPK signaling and decreased the expression of the Wnt inhibitor sclerostin [33]. LRP2 (protein accession number Q4ZG84; uncharacterized; FC = 1.55) improved angiogenesis after treatment with CRA in brain endothelial cells and was an upregulated inflammatory molecule and important receptor for lipocalin 2 [59, 60].

Although the role of the Wnt signaling pathway in CRA-induced VI has not yet been reported, studies have shown that β-catenin-mediated Wnt signaling plays a critical role in cardiac hypertrophy [61], vascular calcification [62], arterial stiffness [63], and angiogenesis [64]. We have determined the expression of 5 indicators related to the Wnt signaling pathway; however, the mechanism underlying the involvement of the Wnt signaling pathway in CRA-induced VI remains to be elucidated. Therefore, we have continued to identify classic indicators involving β-catenin and Wnt3a after treatment with CRA. Activation of the Wnt signaling pathway by CRA is one of the key regulatory systems in coordinating HAEC behavior to induce vascular morphogenesis; particularly, Wnt3a promotes the proliferation, migration, differentiation, and survival of endothelial cells [65].

Our study showed that three differentially expressed proteins were associated with the ErbB signaling pathway (Table 1 and additional file show this in more detail—see Additional files 5 and 6) and thus were used as indicators (Table 1). Arterial dysfunction was confirmed first by the downregulation (FC = 0.45) of protein kinase C (protein accession number B4DFV1), resulting in enhanced Ca^2+ ^-sensing receptor-mediated relaxation of phenylephrine-contracted mesenteric arteries [37]. ErbB results in activation of several signaling pathways that are shared by other tyrosine kinase receptors involved in angiogenesis, such as VEGF receptors [66]. The expression of a protein (FC = 1.65), such as breast cancer anti-estrogen resistance 1 (protein accession number Q96CD4), is associated with elevated vascular endothelial growth factor and p53 expression levels [67]. Upon treatment with CRA, upregulation of RNF115 genes, followed by expression of E3 ubiquitin-protein ligase RNF115 (FC = 16.09) with protein accession number Q9Y4L5, was found to be linked to atherosclerosis pathways [68].

ErbB expression was detected in various endothelial cells, including HAECs. We first elucidated the mechanism of the ErbB signaling pathway in CRA-induced VI. Our results confirmed that CRA directly activates the proteins ErbB2 and ErbB4. Although the role of ErbB signaling in CRA-induced VI remains unclear, studies have shown decreased activation of ErbB2 and inhibition of corneal angiogenesis in response to burn injury [69]. Moreover, high expression levels of NRG/ErbB promote vascular endothelial growth factor (VEGF) release in tumors, which stimulates angiogenesis [70]. ErbB2 and ErbB4 receptors, VEGF receptors, and MAPK pathways are interconnectedly involved in the onset of angiogenesis [66], and the activation of MAPK pathways (Fig. 1E) in this research also suggests that ErbB expression was activated by CRA.

## Conclusions

To the best of our knowledge, our study is the first to demonstrate the mechanism of the Wnt and ErbB signaling pathways in VI development and to manually classify 34 proteins as indicators (Table 1), according to the indicators used to analyze the mechanisms of DNA interstrand crosslinks; GSH perturbation; mitogen-activated protein kinase; and the Wnt and ErbB signaling pathways. Further, we constructed profiles of the mechanism underlying VI-induced by CRA. However, this study has some limitations. First, the 34 identified proteins were determined using HAECs, but were not confirmed in clinical samples; however, these results may guide further clinical studies to explore the mechanisms of these indicators induced by CRA. Second, a limited number of the proteins (Table 1) have been previously listed as diagnostic biomarkers in other studies, which include alpha-2-macroglobulin (protein accession number P01023). However, these studies have either focused on disease factors other than VI, such as the effects of a high salt diet, or have not evaluated CRA exposure as part of the underlying disease mechanisms.

## Supplementary Information

**Additional file 1: Table S1.** Quantitative analysis of total proteins by TMT-based proteomics analysis.

**Additional file 2: Table S2.** Identification of total protein by TMT- based proteomics analysis.

**Additional file 3: Table S3.** Identification of total peptides by TMT-based proteomics analysis.

**Additional file 4: Table S4.** Differentially expressed proteins after treatment with CRA.

**Additional file 5: Table S5.** Classification of each differentially expressed protein according to the GO and KEGG analysis.

**Additional file 6: Table S6.** Functional assays on the identified 34 indicators.

**Additional file 7: Figure S1.** Heatmap of the differentially expressed proteins. Each row represents a different protein, whereas columns represent different samples. Each color represents a different magnitude of expression (log2 expression). Red indicates the higher-level proteins, while blue represents the lower-level proteins.

## Data Availability

The dataset(s) supporting the conclusions of this article is(are) included within the article [and its additional file(s)].
