# Supplementary material for: Proteomics-based evaluation of the mechanism underlying vascular injury via DNA interstrand crosslinks, glutathione perturbation, mitogen-activated protein kinase, and Wnt and ErbB signaling pathways induced by crotonaldehyde
Source: Clin Proteomics. 2022 Aug 24;19:33. doi: 10.1186/s12014-022-09369-7 (PMC9400244; doi:10.1186/s12014-022-09369-7)
Supplement: Supplementary file 5 — Additional file 5: Table S5. Classification of each differentially expressed protein according to the GO and KEGG analysis. [file 12014_2022_9369_MOESM5_ESM.docx]

**Additional file 5-Classification of each differentially expressed protein according to the GO and KEGG analysis.docx**

| GO analysis | Protein group accessions of different proteins (FC>1.5 or <0.667) |
| --- | --- |
| DNA damage and repair | A0A024R2V4, A0A090N8Z2, A0A1W2PQ15, D3DXI9, D6RAJ6, F8WBH5, P01023 (4),P38935, Q5TG38 (1), Q68DE6, Q9BY89 |
| Oxidation-reduction process | A0A090N8Z2, A0A2P9ADI0, B1AH59, B4DRL9, B4DV58, B4DWB3, B4DY35, H0YEL3, H0YL12 (3), O95101 |
| Mitochondrial | A0A2P9AJA0, E7EVY0 (4), P48507 (1) |
| MAPK cascade | B4DI57, J3KND1, M0R366, P19838, Q9UPS8 |
| Arterial dysfunction | B4DFV1,B4DV58, |
| Vascular remodeling | A0A024R2V4, A0A024R8I8, P48507 (1), A0A0S2Z4N4, A0A2P9AEI5, B4DI57, B4DPN0, C9J080, E9PIQ7, E9PP49, H0YBF7 (1), Q8TC04 (1), I3L0X8, J3KND1, M0R366, P02533, P05109 (1), P19532, Q13445, Q13797, Q96PY5 |
| Disintegrity and dysfunction of HAECs | P48507 (1), A0A0S2Z4N4, A0A0X1KG69, C9J080, E9PP49, Q8TC04 (1), J3KND1, P19532, Q13445, Q13797 |
| Lipid metabolism | A0A2P9ADI0, A0A2P9AF66, A0A2P9AJA0, A0A2P9AMK3, B1AH59, B4DJ23, E7ER60, P19838, Q5SXX8, |
| Immunity | A0A1B0GV06 (1), A0A1W2PQ15, B4DFV1, B4DV58, , B4E380, H0YEL3, Q8TC04 (1), M0R2K7, M0R366, P02765 (7), P04080, P05109 (1), P05546 (1), P19532, P19838, Q13797, Q68DE6, Q8N999, Q96CD4, Q9NYJ1, Q9Y2Y1 |
| Autophagy | B4DJ23, P05109 (1) |
| Blood coagulation | A0A087WTU3 (4), B4DI57, B4DPN0, B4E380, H7C4J6, P02765 (7), P05109 (1), P05546 (1), Q13797, Q96CD4, |
| NF-κB signaling pathway | A0A1W2PQ15, B4DDK7, P05109 (1) |
| BMP signaling pathway | A0A2P9AIR3，F5GY03, B3KP05, Q8NC56, Q4ZG84, A0A024R0F6 |
| Wnt signaling pathway | A0A024R2V4, A0A2P9AEI5, F8WBH5, P19838, Q8N7H5, F5GY03, B3KP05, Q8NC56, Q4ZG84, A0A024R0F6 |
| ErbB signaling pathway | B4DFV1, Q96CD4, Q9Y4L5, Q9NYJ1 |
| Apoptosis or cell cycle | A0A1W2PQ15, A0A2P9AJA0, B2RDY3, B4DN11, B4DPN0, B4DRL9, E9PIQ7, F8WBH5, H0YDG7, H3BNV7, H7BZW6, J3KND1, P05109 (1), P19838, P48507 (1), Q68DE6, Q8ND71, Q8TC04 (1), Q96CD4 |

Note: numbers in parentheses represent the number of different nodes connected by the same node.
