# Supplementary material for: Proteomics-based evaluation of the mechanism underlying vascular injury via DNA interstrand crosslinks, glutathione perturbation, mitogen-activated protein kinase, and Wnt and ErbB signaling pathways induced by crotonaldehyde
Source: Clin Proteomics. 2022 Aug 24;19:33. doi: 10.1186/s12014-022-09369-7 (PMC9400244; doi:10.1186/s12014-022-09369-7)
Supplement: Supplementary file 6 — Additional file 6: Table S6. Functional assays on the identified 34 indicators. [file 12014_2022_9369_MOESM6_ESM.docx]

Additional file 6- Functional assays on the identified 34 indicators

| Accession | GO - Molecular function | GO - Biological process |
| --- | --- | --- |
| A0A024R2V4 | GO:0005488,binding | GO:0051716,cellular response to stimulus  GO:0007224,smoothened signaling pathway  GO:0007166,cell surface receptor signaling pathway  GO:0007154,cell communication\| |
| A0A0X1KG69 | GO:0005488,binding  GO:0005515,protein binding | GO:0022607,cellular component assembly  GO:0035556,intracellular signal transduction  GO:0070830,bicellular tight junction assembly |
| A0A1B0GV06 | GO:0016811,hydrolase activity, acting on carbon-nitrogen (but not peptide) bonds, in linear amides\| | GO:0034641,cellular nitrogen compound metabolic process  GO:0006629,lipid metabolic process  GO:0002283,neutrophil activation involved in immune response  GO:0016192,vesicle-mediated transport  GO:0043312,neutrophil degranulation |
| A0A2P9AF66 | GO:0016788,hydrolase activity, acting on ester bonds  GO:0052834,inositol monophosphate phosphatase activity  GO:0042803,protein homodimerization activity | GO:0006650,glycerophospholipid metabolic process  GO:0044283,small molecule biosynthetic process  GO:0006629,lipid metabolic process  GO:0046164,alcohol catabolic process  GO:0005975,carbohydrate metabolic process |
| A0A2P9AJA0 | GO:0004672,protein kinase activity  GO:0043167,ion binding  GO:1901363,heterocyclic compound binding  GO:0001883,purine nucleoside binding  GO:0016773,phosphotransferase activity, alcohol group as accepto  GO:0004740,pyruvate dehydrogenase (acetyl-transferring) kinase activity | GO:0006631,fatty acid metabolic process  GO:0098779,mitophagy in response to mitochondrial depolarization  GO:0044262,cellular carbohydrate metabolic process  GO:0097411,hypoxia-inducible factor-1alpha signaling pathway  GO:0008631,intrinsic apoptotic signaling pathway in response to oxidative stress  GO:0010510,regulation of acetyl-CoA biosynthetic process from pyruvate |
| B4DFV1 | GO:0004672,protein kinase activity  GO:0043167,ion binding | GO:0004912,GnRH signaling pathway  GO:0004930,Type II diabetes mellitus  GO:0004530,Tight junction  GO:0004722,Neurotrophin signaling pathway  GO:0004750,Inflammatory mediator regulation of TRP channels  GO:0038127,ERBB signaling pathway  GO:0004915,Estrogen signaling pathway  GO:0004933,AGE-RAGE signaling pathway in diabetic complications  GO:0004062,Chemokine signaling pathway  GO:0004931,Insulin resistance  GO:0004270,Vascular smooth muscle contraction |
| B4DI57 | GO:1990459,transferrin receptor binding  GO:0046914,transition metal ion binding  GO:0072510,trivalent inorganic cation transmembrane transporter activity | GO:0001895,retina homeostasis  GO:0007596,blood coagulation  GO:0007178,transmembrane receptor protein serine/threonine kinase signaling pathway  GO:0060395,SMAD protein signal transduction  GO:0006812,cation transpor  GO:0042060,wound healing  GO:0043410,positive regulation of MAPK cascade  GO:0034105,positive regulation of tissue remodeling |
| B4DJ23 | GO:0052866,phosphatidylinositol phosphate phosphatase activity  GO:0004725,protein tyrosine phosphatase activity | GO:0006650,glycerophospholipid metabolic process  GO:0044267,cellular protein metabolic process  GO:0006914,autophagy |
| B4DPN0 | GO:1901681,sulfur compound binding  GO:0043168,anion binding  GO:0005543,phospholipid binding  GO:0097367,carbohydrate derivative binding  GO:0030234,enzyme regulator activity  GO:0060230,lipoprotein lipase activator activity  GO:0008201,heparin binding  GO:0005539,glycosaminoglycan binding | GO:0050680,negative regulation of epithelial cell proliferation  GO:0072358,cardiovascular system development  GO:0010633,negative regulation of epithelial cell migration  GO:0010656,negative regulation of muscle cell apoptotic process  GO:0034392,negative regulation of smooth muscle cell apoptotic process  GO:0051918,negative regulation of fibrinolysis  GO:0072378,blood coagulation, fibrin clot formation |
| B4DV58 | GO:0052869,heme binding  GO:0005488,iron ion binding  GO:1901363,oxidoreductase activity, acting on paired donors, with incorporation or reduction of molecular | GO:0051047,positive regulation of secretion  GO:0098771,inorganic ion homeostasis  GO:0097267,omega-hydroxylase P450 pathway  GO:0072329,monocarboxylic acid catabolic process  GO:0042758,long-chain fatty acid catabolic process  GO:0055114,oxidation-reduction process  GO:0003073,regulation of systemic arterial blood pressure  GO:0036102,leukotriene B4 metabolic process |
| B4DWB3 | GO:0016818,hydrolase activity, acting on acid anhydrides, in phosphorus-containing anhydrides  GO:0022892,substrate-specific transporter activity  GO:0015278,calcium-release channel activity  GO:0005217,intracellular ligand-gated ion channel activity | GO:0055114,oxidation-reduction process |
| C9J080 | GO:0019901,protein kinase binding  GO:0005200,structural constituent of cytoskeleton  GO:0005516,calmodulin binding | GO:0042493,response to drug  GO:0055085,transmembrane transport  GO:0007010,cytoskeleton organization  GO:0071840,cellular component organization or biogenesis |
| D3DXI9 | GO:0034061,DNA polymerase activity  GO:0008270,zinc ion binding  GO:0003676,DNA replication  GO:0043167DNA repair | GO:0006289,nucleotide-excision repair  GO:0044843,cell cycle G1/S phase transition  GO:0000731,DNA synthesis involved in DNA repair |
| E7EVY0 | GO:0097177,mitochondrial ribosome binding  GO:0042803,protein homodimerization activity | GO:0033615,mitochondrial proton-transporting ATP synthase complex assembly  GO:0051341,regulation of oxidoreductase activity  GO:0010257,NADH dehydrogenase complex assembly |
| E9PP49 | GO:0030020,extracellular matrix structural constituent conferring tensile strength | GO:0016337,single organismal cell-cell adhesion  GO:0048514,blood vessel morphogenesis |
| F8WBH5 | GO:0042393,histone binding  GO:0030234,enzyme regulator activit  GO:0032403,protein complex binding  GO:0044877,macromolecular complex binding | GO:0060548,negative regulation of cell deat  GO:0002429,immune response-activating cell surface receptor signaling pathway  GO:0090090,negative regulation of canonical Wnt signaling pathway  Pathway  GO:0030330,DNA damage response, signal transduction by p53 class mediatodeubiquitinatio |
| H0YEL3 | GO:0044769, hydrolase activity,ATPase activity, coupled to transmembrane movement of ions, rotational mechanism | GO:0007169,transmembrane receptor protein tyrosine kinase signaling pathway e-triphosphatase activity  GO:0046961,proton-transporting ATPase activity, rotational mechanism |
| H0YL12 | GO:0043168,anion binding  GO:0050662,coenzyme binding  GO:0000166,nucleotide binding  GO:0009055,electron carrier activity  GO:1901363,heterocyclic compound binding | GO:0015980,energy derivation by oxidation of organic compounds  GO:0044699,single-organism process  GO:0055114,oxidation-reduction process  GO:0033539,fatty acid beta-oxidation using acyl-CoA dehydrogenase |
| O95101 | GO:0004129,cytochrome-c oxidase activity  GO:0008324,cation transmembrane transporter activity  GO:0016676,oxidoreductase activity, acting on a heme group of donors, oxygen as acceptor | GO:0042775,mitochondrial ATP synthesis coupled electron transport  GO:0006796,phosphate-containing compound metabolic process  GO:1901135,carbohydrate derivative metabolic process |
| P01023 | GO:0030414,peptidase inhibitor activity  GO:0048306,calcium-dependent protein binding  GO:0019959,interleukin-8 binding  GO:0019838,growth factor binding  GO:0043120,tumor necrosis factor binding  GO:0005096,GTPase activator activity | GO:0050776,regulation of immune response  GO:0030168,platelet activation  GO:0030154,cell differentiation  GO:0009611,response to wounding  GO:0072378,blood coagulation, fibrin clot formation  GO:0043547,positive regulation of GTPase activity  GO:0051345,positive regulation of hydrolase activity |
| P02765 | GO:0008565,protein transporter activity  GO:0019901,protein kinase binding  GO:0015197,peptide transporter activity | GO:0002682,regulation of immune system process  GO:0007507,heart development  GO:0048856,anatomical structure development  GO:0015833,peptide transport  GO:0009855,determination of bilateral symmetry |
| P05109 | GO:0050786,RAGE receptor binding  GO:0044877,macromolecular complex binding  GO:0008270,zinc ion binding  GO:0036041,long-chain fatty acid binding  GO:0043169,cation binding  GO:0035662,Toll-like receptor 4 binding  GO:0008092,cytoskeletal protein binding | GO:2001233,regulation of apoptotic signaling pathway  GO:0048870,cell motility  GO:0043068,positive regulation of programmed cell death  GO:0002376,immune system process  GO:0035556,intracellular signal transduction  GO:0098609,cell-cell adhesion  GO:0050790,regulation of catalytic activity  GO:0042060,wound healing  GO:0051092,positive regulation of NF-kappaB transcription factor activity  GO:0006914,autophagyGO:0007010,cytoskeleton organization |
| P05546 | GO:1901681,sulfur compound binding  GO:0008201,heparin binding  GO:0005539,glycosaminoglycan binding  GO:0004867,serine-type endopeptidase inhibitor activity | GO:0009611,response to wounding  GO:0007596,blood coagulation  GO:0043412,macromolecule modification |
| P19838 | GO:0031072,heat shock protein binding  GO:0001158,enhancer sequence-specific DNA binding  GO:0008092,cytoskeletal protein binding | GO:0090263,positive regulation of canonical Wnt signaling pathway  GO:0051341,regulation of oxidoreductase activity  GO:0002684,positive regulation of immune system process  GO:0008219,cell death  GO:0042359,vitamin D metabolic process  GO:0033993,response to lipid  GO:0090077,foam cell differentiation |
| P48507 | GO:0046982,protein heterodimerization activity  GO:0003824,catalytic activity  GO:0035226,glutamate-cysteine ligase catalytic subunit binding  GO:0016879,ligase activity, forming carbon-nitrogen bonds | GO:2001233,regulation of apoptotic signaling pathway  GO:0032844,regulation of homeostatic process  GO:0003018,vascular process in circulatory system  GO:0007005,mitochondrion organization  GO:0009064,glutamine family amino acid metabolic process  GO:1990823,response to leukemia inhibitory factor |
| Q13797 | GO:0043169,cation binding  GO:0043167,ion binding  GO:1990405,protein antigen binding  GO:0050839,cell adhesion molecule binding  GO:0001968,fibronectin binding  GO:0038023,signaling receptor activity  GO:0019960,C-X3-C chemokine bindin  GO:0019955,cytokine binding | GO:0034113,heterotypic cell-cell adhesion  GO:0007596,blood coagulation  GO:0002376,immune system process  GO:0000904,cell morphogenesis involved in differentiation  GO:0061458,reproductive system development  GO:0022407,regulation of cell-cell adhesion  GO:0043067,regulation of programmed cell death |
| Q4ZG84 | GO:0005488,binding  GO:0042813,Wnt-activated receptor activity  GO:0005041,low-density lipoprotein receptor activity  GO:0031994,insulin-like growth factor I binding | GO:0006775,fat-soluble vitamin metabolic process  GO:0044332,Wnt signaling pathway involved in dorsal/ventral axis specification  GO:0072358,cardiovascular system development  GO:0030509,BMP signaling pathway  GO:0007178,transmembrane receptor protein serine/threonine kinase signaling pathway  GO:0046660,female sex differentiation  GO:0043066,negative regulation of apoptotic process |
| Q5TG38 | GO:0001085,RNA polymerase II transcription factor bindingGO:0005515,protein binding  GO:0001071,nucleic acid binding transcription factor activity | GO:0033157,regulation of intracellular protein transport  GO:0006475,internal protein amino acid acetylation  GO:0006306,DNA methylation  GO:0097305,response to alcohol  GO:0006259,DNA metabolic process |
| Q8N7H5 | GO:0005102,receptor binding  GO:0005515,protein binding  GO:0001664,G-protein coupled receptor binding  GO:0004857,enzyme inhibitor activity  GO:0098772,molecular function regulator | GO:0032147,activation of protein kinase activityGO:0098779,mitophagy in response to mitochondrial depolarizationGO:0060326,cell chemotaxis\|GO:0002455,humoral immune response mediated by circulating immunoglobulinGO:0000165,MAPK cascadeGO:0048514,blood vessel morphogenesisGO:0071902,positive regulation of protein serine/threonine kinase activityGO:0043405,regulation of MAP kinase activity |
| Q8TC04 | GO:0005200,structural constituent of cytoskeleton  GO:0005488,binding | GO:0030154,cell differentiation， morphogenesis of an epithelium  GO:0045087,innate immune response\|  GO:0007568,aging  GO:0012501,programmed cell death\| |
| Q9BY89 | GO:0008092,cytoskeletal protein binding  GO:0030506,ankyrin binding  GO:0045296,cadherin binding  GO:0019904,protein domain specific binding  GO:0050839,cell adhesion molecule binding | GO:0006302,double-strand break repair  GO:0071479,cellular response to ionizing radiation  GO:0006977,DNA damage response, signal transduction by p53 class mediator resulting in cell cycle arrest  GO:0072431,signal transduction involved in mitotic G1 DNA damage checkpoint |
| Q96CD4 | GO:0005515,protein binding  GO:0019900,kinase binding  GO:0060089,molecular transducer activity | GO:0007596,blood coagulation  GO:0006935,chemotaxis  GO:0002429,immune response-activating cell surface receptor signaling pathway  GO:0007173,epidermal growth factor receptor signaling pathway  GO:0042981,regulation of apoptotic process \|GO:0048010,vascular endothelial growth factor receptor signaling pathway  GO:0038127,ERBB signaling pathway \|GO:0007155,cell adhesion |
| Q9Y4L5 | GO:0046914,transition metal ion binding  GO:0005154,epidermal growth factor receptor binding  GO:0016874,ligase activity | GO:1901184,regulation of ERBB signaling pathway  GO:0007173,epidermal growth factor receptor signaling pathway  GO:0044765,single-organism transport  GO:1901564,organonitrogen compound metabolic process  GO:0006807,nitrogen compound metabolic process  GO:0016482,cytosolic transport |
| Q9UPS8 | GO:0045599, negative regulation of fat cell differentiation | GO:0007169,transmembrane receptor protein tyrosine kinase signaling pathway\|  GO:1900077,negative regulation of cellular response to insulin stimulus  GO:0006631,fatty acid metabolic process  GO:0043409,negative regulation of MAPK cascade |
