# Supplementary figures and images for: Proteomics-based evaluation of the mechanism underlying vascular injury via DNA interstrand crosslinks, glutathione perturbation, mitogen-activated protein kinase, and Wnt and ErbB signaling pathways induced by crotonaldehyde
Source: Clin Proteomics. 2022 Aug 24;19:33. doi: 10.1186/s12014-022-09369-7 (PMC9400244; doi:10.1186/s12014-022-09369-7)

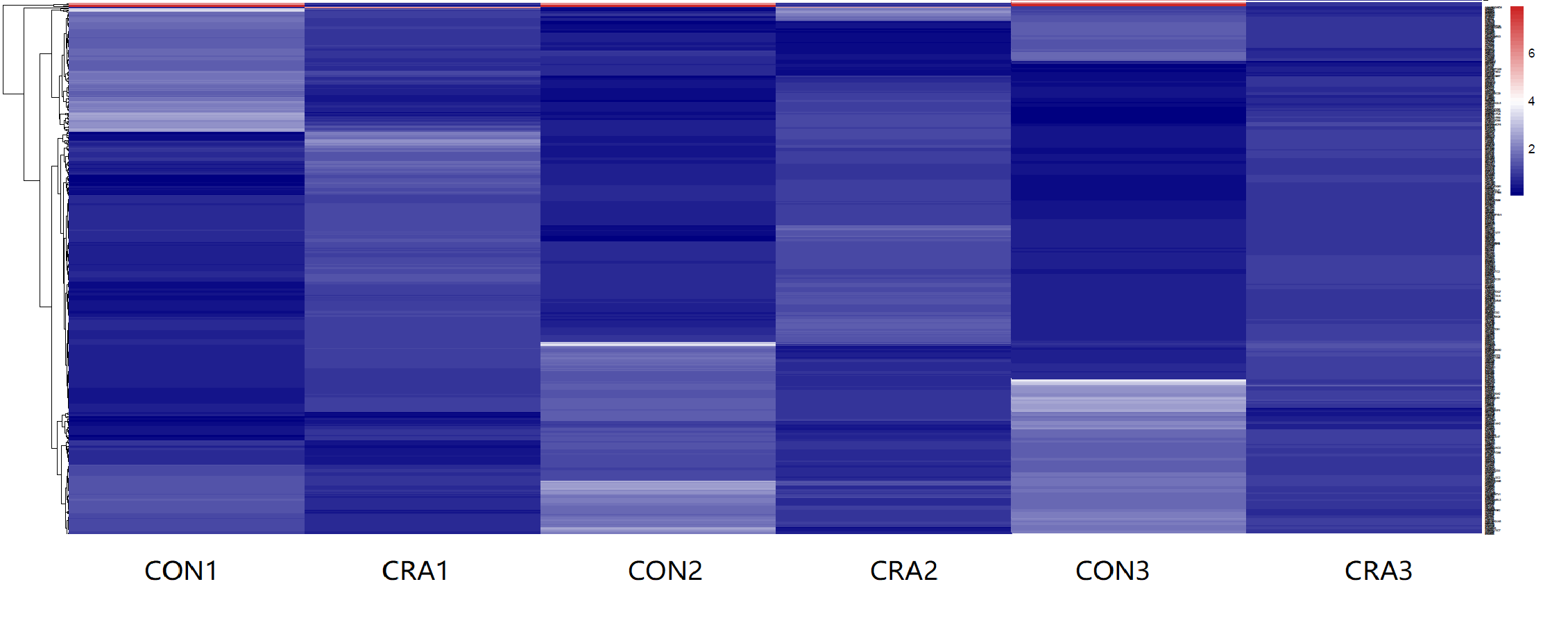

Supplement: Supplementary file 7 — Additional file 7: Figure S1. Heatmap of the differentially expressed proteins. Each row represents a different protein, whereas columns represent different samples. Each color represents a different magnitude of expression (log2 expression). Red indicates the higher-level proteins, while blue represents the lower-level proteins. [file 12014_2022_9369_MOESM7_ESM.png]
